# The Alien Limb Phenomenon in Creutzfeldt-Jakob Disease: A Systematic Review of Case Reports

**DOI:** 10.7759/cureus.27029

**Published:** 2022-07-19

**Authors:** Meghdeep Sen, Juan A Moncayo, Maria A Kelley, Deydie Suarez Salazar, Maria Gabriela Tenemaza, Mikaela Camacho, Gashaw Hassen, Guillermo E Lopez, Gustavo Monteros, Gabriela Garofalo, Ayush Yadav, Juan Fernando Ortiz

**Affiliations:** 1 Medicine, American University of Antigua, St. John's, ATG; 2 Neurology, Pontificia Universidad Católica del Ecuador, Quito, ECU; 3 General Medicine, Universidad de Guayaquil, Guayaquil, ECU; 4 Emergency, Hospital Oskar Jandl, Galapagos, ECU; 5 Medicine, Universidad San Francisco de Quito, Quito, ECU; 6 Neurology, Universidad San Francisco De Quito, Quito, ECU; 7 Internal Medicine, Mercy Medical Center, Baltimore, USA; 8 Medicine, Universidad de Cuenca, Cuenca, ECU; 9 General Medicine, Pontificia Universidad Católica del Ecuador, Quito, ECU; 10 Medicine, Universidad Central Del Ecuador, Quito, ECU; 11 Medicine, University of Antigua, St. John's, ATG; 12 Neurology, Universidad San Francisco de Quito, Quito, ECU

**Keywords:** callosal phenotype, anterior phenotype, posterior phenotype, creutzfeldt-jakob disease, alien limb phenomenon

## Abstract

Alien limb phenomenon (ALP) is a clinical finding seen in numerous neurological disorders, including Creutzfeldt-Jakob disease (CJD). We aimed to conduct a systematic review to update advances in understanding the classification and pathophysiology of ALP in CJD. We used PubMed advanced-strategy searches and only included full-text observational studies and case reports conducted on humans and written in English. We used the PRISMA protocol for this systematic review and the Methodological Quality of Case Reports tool to assess the bias encountered in each study. After applying the inclusion/exclusion criteria, 10 case reports were reviewed. Two independent reviewers analyzed data and confirmed the phenotype of each case of the alien limb in CJD separately. Overall, the most prevalent ALP phenotype presenting in patients with CJD was the posterior phenotype, usually in the early stages of the disease. Our findings corroborate previous research in demonstrating the pathophysiology behind ALP in CJD. We suggest physicians suspect CJD whenever patients present with ALP as the initial symptom.

## Introduction and background

The alien limb refers to an extremity with uncontrollable and involuntary motor activity. Goldstein, in 1908, reported the first case in a right-handed woman who had previously suffered a cerebral stroke. The patient complained of a “strange” feeling in her left hand and as if it was being used by someone else. This feeling of strangeness was further investigated by Brion et al. in 1972, who found callosal disconnection to be the underlying cause and called it “The Strange Hand” [1]. Bogen introduced the term “Alien” in 1979, broadening the definition of the condition to include unwanted activities and finally rendering it as an action. While alien limb phenomenon (ALP) has primarily been reported in the upper limbs, lower limbs can also be affected [2]. Research over the last couple of decades has revealed that the phenomenon is associated with various conditions, including Corticobasal syndrome (CBS), Creutzfeldt-Jakob disease (CJD), Alzheimer's disease, and stroke. In addition, unusual disorders were documented as possible causes of ALP, such as demyelinating disease, progressive multifocal leukoencephalopathy, hereditary diffuse leukoencephalopathy with spheroids, posterior reversible encephalopathy syndrome, corpus callosotomy, thalamic dementia, intracerebral hemorrhage, and thalamic dementia [2,3].

Previous reports have focused on ALP as a consequence of lesions located in the corpus callosum associated with ischemic or hemorrhagic anterior cerebral artery stroke and possible involvement of medial frontal structures [3]. With more published manuscripts, ALP is now known to present several clinical phenotypes based on lesion localization, mainly as anterior and posterior phenotypes. The posterior phenotype is predominantly seen in CBS [4-6] but has also been reported in CJD and stroke [7,8]. This variation has a diversity of features such as apraxia, neglect, rigidity, cortical sensory findings, and noticeable estrangement from the limb [5,6]. The involvement of the corpus callosum and the frontal lobes were principally affected in CBS with abnormal posturing and limb elevation presentation [2]. In some studies, about 50% of CBS patients described the symptoms as “tend to hold the offending hand with the better hand’’ with an “unwilled arm levitation’’ and/or “sensation of foreignness of the limb’’ [9]. There was no association between ALP and limb apraxia severity. It is difficult to tell whether individuals with apraxia also have levitation and gripping problems or if patients with posterior ALP have cortical sensory symptoms [10-12].

CJD is a rare but lethal neurodegenerative disease that is characterized by myoclonus, ataxia, rapidly progressive dementia, visual disorders, kinetic mutism along with pyramidal and extrapyramidal symptoms [13]. The abnormal buildup of aberrant prion protein (PrP), neuronal death, and spongiform alteration throughout the brain cause CJD. The disease can be sporadic (sCJD), familial (fCJD), iatrogenic (iCJD), or variant (vCJD), in which PrP is passed to humans across a species barrier. CJD has a variety of clinical and pathological manifestations due to its many etiologies [14]. The commonly affected brain regions are the basal ganglia, cingulate gyrus, and occipital cortical areas [7,13]. Some reports describe the presence of ALP as a clinically significant finding, prompting a search for the underlying pathophysiology. ALP and unilateral spatial neglect (USN) are uncommon CJD symptoms that might be difficult to detect. Zannino et al. described two cases of sCJD with USN and ALP as noticeable clinical features. ALP can be a unique and important characteristic of CJD, particularly in the early stages. The basic neuropathological and neuroradiological abnormalities of CJD are hyperintensities and limited diffusion in cortical or basal ganglia regions, as shown on FLAIR/DWI. Neglect symptoms have been reported following damage to several brain regions, including the putamen, caudate, pulvinar, and parietal-frontal cortex. In contrast, USN is generally associated with pathological processes involving the right hemisphere. Injury to the posterior frontal and superior parietal lobes have been known to cause ALP, particularly the posterior phenotype of ALP. As a result, USN and ALP are clinical symptoms that develop as a result of injury to brain regions implicated in the neuropathological process of CJD [7,15].

Only a few articles have been written regarding the phenotype of the alien limb in CJD. A systematic review of the topic has not yet been written in the literature about the ALP associated with CJD and its clinical phenotypes. We aim to compare and contrast different observational studies and case reports and define the prevalence of the different phenotypes of ALP in CJD and investigate their pathophysiology, which remains poorly understood.

## Review

Methods

Protocol

We carried out a systematic review using the PRISMA protocol for this systematic review.

Eligibility Criteria and Study Selection

We included only case reports conducted on humans and written in the English Language. We excluded systematic reviews, literature reviews, and Meta-Analyses. We selected studies according to the following criteria: (1) Patients: Variant CJD; (2) Intervention: Patients with ALP (3) Comparator: No Comparison, and (4) Outcomes: Evaluate the prevalence and phenotype of the ALP variant in patients with CJD.

Database and Search Strategy

We used the PubMed database for this systematic review. The search did not have a time frame due to the rare frequency of cases of CJD and ALP. We used the following terms to conduct the search: (“creutzfeldt Jakob disease”[Title/Abstract] AND “alien limb”[Title/Abstract]) OR (“creutzfeldt Jakob disease”[Title/Abstract] AND “alien hand”[Title/Abstract])

Data Extraction and Analysis

We collected the following information from each paper: Author, year, clinical features, MRI, EEG, and Alien limb/hand Phenotype. The phenotype of alien limb/hand in the cases was analyzed and correlated with the MRI findings in each case.

Bias Assessment

We used the Methodological Quality of Case Reports Tool proposed by Murad et al. and gave punctuation for every item met and later, we did an overall judgement of the methodological quality based on the most critical questions [16]. Table 1 shows the BIAS analysis of this paper using the methodological quality of the case report tool [7,17-25].

**Table 1:** Methodological quality of case reports tool

STUDY	METHODOLOGICAL QUALITY OF CASE REPORTS TOOL				RISK OF BIAS
	Selection (1*)	Ascertainment (max 2*)	Causality (max 4*)	Reporting (1*)	
Ciarlariello et al., Brazil, 2018 [17]	*	**	**	*	Low
Gonzales-Martinez et al., Spain, 2020 [18]	*	**	**	*	Low
Fogel et al., USA, 2006 [19]	*	**	*	*	Moderate
Oberndorfer et al., Austria, 2001 [20]	*	*	*	*	High
Rabinstein et al., USA, 2002 [21]	*	**	**	*	Low
Inzelberg et al., Israel, 2000 [22]	*	*	*	*	Moderate
Macgowan et al., USA, 1997 (Case 1) [23]	*	**	**	*	Low
Macgowan et al., USA, 1997 (Case 2) [23]	*	**	**	*	Low
Avanzino et al., Italy, 2005 [24]	*	**	**	*	Low
Kleiner-Fisman et al., Canada, 2004 [25]	*	*	**	*	Low
Rubin et al., USA, 2012 [7]	*	**	**	*	Low

Results

**Figure 1:**
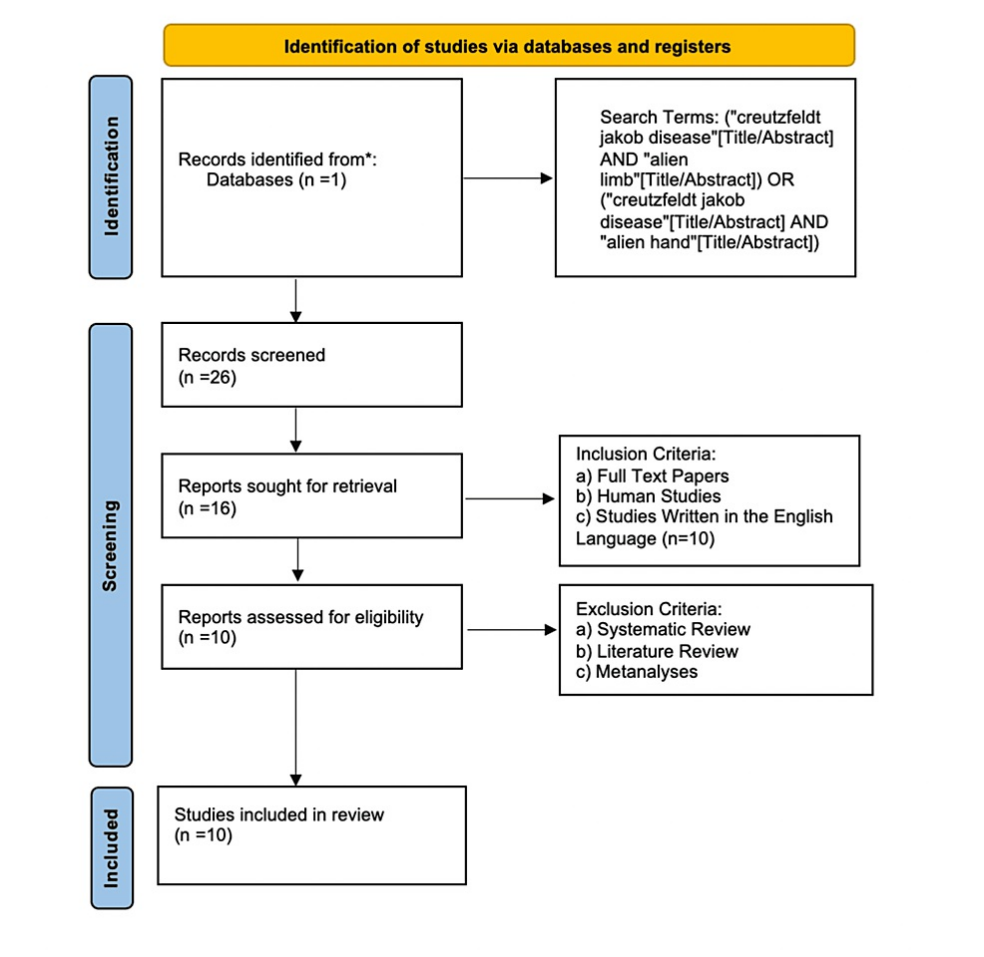
PRISMA flowchart of the systematic review

Figure 1 shows the PRISMA flowchart of this systematic review.

Table 2 shows the clinical, EEG, imaging , and phenotype features of the cases of ALP and CJD [7,17-25].

**Table 2:** Study characteristics. EEG: electroencephalogram, LPD: lateralized periodic discharges, PSWC: periodic sharp waves complexes, DWI: diffuse-weighted imaging, MRI: magnetic resonance imaging, HMF: higher mental functions, MMT: manual muscle testing

Author, Year, Country	Clinical Features	EEG	MRI/CT	Alien Hand/Limb Features	Phenotype
Ciarlariello et al., Brazil, 2018 [17]	Mental Status: Gait/Cerebellar: Sensory: Motor	Diffuse delta activity, evolving to lateralized (right side) and diffuse periodic sharp waves complexes (PSWC)	DWI: Diffusion restriction, predominantly in the right parietal cortex (cortical ribbon sign), caudate, and putamen basal ganglia	Levitation of the left arm	Posterior
Gonzales-Martinez et al., Spain, 2020 [18]	Mental Status: Cognitive Impairment, Impaired Consciousness, Fluctuating Aphasia, Bradypsychia. Gait/Cerebellar: Normal Sensory: Normal Motor: Multisegmentary Myoclonus, Hyperreflexia	Routine EEG showed PSWC/ LPD with a medium amplitude and frequency of >2Hz.	T2: Hyperintensities: right cerebral cortex and basal ganglia. DWI: basal ganglia/cortical hyperintensity	Levitation of the arm	Posterior
Fogel et al., USA, 2006 [19]	Mental Status: Oriented and Alert. Gait/Cerebellar: Repeat falls, ataxic gait Sensory: Paraesthesia in the left-hand Motor: Jerking movement/ myoclonus in upper extremities, dystonic Posturing, apraxia in both hands, increased reflexes, Positive Babinski on the left side.	PSWC	DWI: restricted diffusion within the cortex involving the parietal, occipital, and posterior temporal lobes bilaterally but greater on the left, as well as the anterior left temporal, insular cortex, and the left frontal lobe FLAIR: correlation with DWI, and no postcontrast enhancement	Hand Interference and Grasping	Frontal/Callosal
Oberndorfer et al., Austria, 2001 [20]	Mental status: Alert and Oriented Gait/Cerebellar: Ataxia in left-hand Sensory: No sensory loss Motor: Mild distal paresis, positive grasp reflex. Spontaneous athetoid like movements of the left hand, increased reflexes, positive Babinski	Multilocular periodic triphasic waveforms	MRI: Multiple periventricular subcortical confluent localized hyperintense lesions in T2	Grasping	Anterior
Rabinstein et al., USA, 2002 [21]	Mental Status: Stuporous and during wake mute and abulic Gait/Cerebellar: Not specified Sensory: Astereognosis Motor: Bilateral rigidity and hyperreflexia. Involuntary movements of the right hand, followed by akinetic mutism and myoclonic movements in the upper extremities. Left-hand apraxia and jerky movements in the right arm	Bilateral independent spikes and sharp waves superimposed on a slow, disorganized background; Epileptiform prominent in the right hemisphere.	DWI: Restricted diffusion in bilateral parietal, occipital cortices, and frontotemporal lobes; an abnormal signal was seen in putamina bilaterally and right caudate head MRI: Hyperintensity in the parieto-occipital regions bilaterally	Bilateral Hand interference	Callosal
Inzelberg et al., Israel, 2000 [22]	Mental Status: Awake, bradypnea, disoriented, able to follow oral instructions. HMF: dysgraphia and dyscalculia, immediate and short-term memory disturbed but long-term spared Gait/Cerebellar: Unsteady gait and frequent falls. Sensory: Normal Motor: Normal force, deep reflexes symmetric, plantar reflex flexor, dystonic posture in the right arm.	Triphasic waves with a periodic pattern of 1- 1.5 Hz.	CT: Mild cerebral atrophy.	Left Arm Levitation	Posterior
Macgowan et al., USA, 1997; Case 1 [23]	Mental status: awake, attentive, oriented MMT: normal gait/cerebellar: ataxia Sensory: severe left-sided sensory disturbance Motor: left spastic hemiparesis, myoclonus.	Periodic complex with triphasic waves of 1 Hz	MRI: Uninformative	Hand interference	Callosal
Macgowan et al., USA, 1997; Case 2 [23]	Mental Status: Oriented MMT: normal Gait/cerebellar: sensory ataxia Sensory: Intact responses to pinprick bilaterally but with alloaesthesia on the left arm. Proprioception was absent in the left arm and leg and absent to the knee and elbow on the right Motor: myoclonus in both hands left more than right, grasp and palmomental reflex present, supranuclear left facial and palatal weakness, spastic tetraparesis affecting the left side more than the right	Low amplitude background with delta slowing and generalized periodic 1-2Hz complexes.	MRI: Uninformative	Grasping	Anterior
Avanzino et al., Italy, 2005 [24]	Mental status: oriented, mildly bradyphrenic MMT: gait/cerebellar: ataxic gait Sensory: normal Motor: oculomotor apraxia (both horizontal and vertical) and dysarthria; left-side pyramidal signs with mild rigidity and bradykinesia were also present	Triphasic waves with a periodic pattern of 1-1.5hz	CT: Normal MRI: Normal	Left-arm levitation	Posterior
Kleiner-Fisman et al., Canada, 2004 [25]	Mental Status: Disoriented to time. Gait/Cerebellar: Ataxia and wide-based gait. Sensory: Normal Motor: left-sided apraxia, deep tendon reflexes, and tone increased, dysphagia, aphasia, incontinence occurred eventually	Sharp and triphasic waves over right frontotemporal lobes	DWI: Signal abnormality in the right caudate head as well as gyriform enhancement, right greater than left, in both cerebral hemispheres	Levitation of left arm and leg	Posterior
Rubin et al., USA, 2012 [7]	Mental status: mild cognitive disturbance MMT: gait/cerebellar: ataxic gait Sensory: left-sided cortical sensory loss Motor: ataxia, left hemineglect optic ataxia, oculomotor apraxia, simultanagnosia (Balint Syndrome )	-	MRI and FLAIR: Changes in several cortical regions and in the corpora striatum bilaterally DWI: Multifocal but predominantly right parietal restricted diffusion of the cortical ribbon and basal ganglia	Grasping	Anterior

After reviewing all 11, cases we find five cases with posterior phenotype, three patients with anterior phenotype, two patients with callosal phenotype, and one with mix callosal/anterior phenotype.

Discussion

The most common phenotype found in this systematic review was the posterior phenotype. Our finding concord with a study by Rubin et al. [26], which documented 13 cases of ALP with CJD; in that study the more common phenotype was the posterior. We only use the information of one case of Rubin et al., case series, because only was case was fully described, in the rest the explanations were brief. We will discuss each phenotype in the discussion, and the main findings related to these phenotypes in this review.

Posterior Phenotype

Levine et al. reported the first case in 1986 [27]. In the ALP, there is prominent estrangement from the limb Particularly. The posterior phenotype is characterized by uncoordinated hand movements and involuntary levitation of the extremity. The posterior ALP may also coexist with rigidity, apraxia, cortical sensory findings, and neglect. When the thalamus is involved, it leads to cerebellar and sensory ataxia. Additionally, a posterior infarct may cause optic ataxia [2,28].

The “posterior” variant was mainly associated with lesions in the thalamus, the posterior part of the parietal lobe, and the occipital lobe [8]. The parietal lobe seems to be affected in the posterior variant. The parietal lobe provides information about an item's size, shape, distance, and direction. Both the superior and inferior parietal lobules are active during synchronized hand motions. The inferior parietal lobule is a multimodal association region that takes input from various sources, including the main somatosensory and prefrontal cortices. By interfering with typical reactions to extrapersonal space, disrupting these complicated networks between the lobules may produce ALP [2,23].

Patients with CBS typically have parietal disease, and limb apraxia, cortical sensory abnormalities, or ALP as common symptoms. The white matter lesions in these individuals are always contralateral to the afflicted hand. The severance of the parietal lobe from motor centers, likely results in misperception by the other hemispheres, resulting in the sense of loss of awareness of movement [2].

Other studies have shown that both the anomalous feelings and motions of an alien limb are caused by the posterior lip of the post-central gyrus, which is involved in stereognosis [26]. Moreover, the cortical-striatal-thalamic circuitry may be involved, resulting in posterior ALP, and additionally causing sensory symptoms.

According to Zannino et al., the significance of frontoparietal cortex and basal ganglia injuries in the development of ALP in our two instances was validated by MRI and PET data [15]. ALP with subacute neglect syndrome may be an early warning indication of CJD, allowing us to fine-tune the characterization of clinical CJD symptoms [7,15]. Five out of 11 patients in our systematic review were classified as having the posterior ALP with regard to their respective clinical features. EEG results of three out of the five patients revealed a characteristic of triphasic waves. MRI findings differed in all five patients, suggesting that MRI findings may not explain the pathophysiology in the posterior phenotype due to the variability of structures involved in each case.

Anterior Phenotype

The anterior variant more commonly affects the dominant hand (right hand). It is characterized by compulsive grabbing of objects, difficulty in releasing objects when they are taken, grasping, and impulsive groping [29]. In this variant, the patient is aware of belonging to the arm but cannot suppress the hand's movements. The interannual conflict is more seen when there is a lesion in the corpus callosum. Still, it can be present due to clinical interpose.

The anatomical part affected is usually in the area of the medial frontal lobe (supplementary motor cortex, medial prefrontal cortex). The corpus callosum may be involved as well. The most common etiology is a stroke of the anterior communicating artery territory [30]. In the frontal variant, the supplementary motor area, cingulate cortex, and prefrontal cortex have been reported to be involved. The supplementary motor area (SMA) integrates information from the cerebral cortex and subcortical motor structures. For example, the primary motor cortex, pre-motor cortex, and subcortical motor areas. It is hypothesized that lesions to the SMA may result in the release of the normal inhibitory control in the motor cortex [28]. An anterior cerebral artery occlusion might generate a mixed callosal and anterior phenotype pattern, as it involves the corpus callosum and the frontal lobe in this review. In our case series, we find three cases of the anterior phenotype [28,29]. The cases were not associated particularly with MRI or EEG findings.

Callosal Phenotype

The corpus callosum is involved in communication between hemispheres and inhibition of impulses. Disconnection syndromes are frequent when a callosal lesion occurs, especially after callesectomies due to epilepsy surgery [30]. The alien hand or ALP is another type of disconnection syndrome that occurs when the corpus callosum is compromised [30].

The callosal variant affects the non-dominant hand and is characterized by inter manual conflict. The inter-manual conflict comprises hand interference with the contralateral activity of the unaffected limb [31]. A callosal lesion would cause a “disconnection” between both hands, causing errors in the natural action of the hand [32]. The disconnection results in an antagonistic but not disassociated action of the hand. The antagonistic action seems to come from the right hemisphere (non-dominant side). It follows the action of the left hemisphere in an antagonist fashion [30].

Additionally, the callosal variant presents without weakness, or frontal signs previously described [31]. Other symptoms that may be encountered are neglect, alexia, anomia, visual anomia, apraxia, and tactile anomia [31]. The more common etiologies are callosectomy, callosal infarct, or callosal demyelination [29]. Our review found two cases that were not associated with EEG or MRI findings.

Limitations

The major limitation of this systematic review is the limited number of cases. We found 24 cases of ALS associated with CJD, but only in 10 patients were the phenotype described. Another limitation is the complex pathophysiology of CJD. It is challenging to explain why CJD may cause alien limb syndrome. More cases are needed to determine if the alien limb syndrome in CJD had some associations, such as triphasic waves or a distinctive pattern on MRI.

## Conclusions

The ALP is a clinical finding usually seen in CJD. Its pathophysiology is probably due to lesions in areas of the brain that play roles in interconnected circuits, such as lesions in the corpus callosum. Two different phenotypes have been described till date: anterior and posterior. The most prevalent phenotype encountered in CJD was the posterior phenotype, which is usually present in the early stages of the disease, making it a significant clinical finding to consider when evaluating patients with suggestive symptoms of CJD. We report five patients with the posterior phenotype of ALP with a characteristic EEG report of triphasic waves, three with the anterior phenotype, and two with the callosal phenotype. MRI findings in all cases suggest that a complex interplay between the anatomical structures involved may play a role in the underlying pathophysiology of ALP in CJD. The network inside the nervous system is complex, and a disease affecting multiple locations can vary between patients. There is a probability that there are more cases or more phenotypes that haven’t been reported yet.
